# Case report: Psychosis and catatonia in an adolescent patient with adipsic hypernatremia and autoantibodies against the subfornical organ

**DOI:** 10.3389/fpsyt.2023.1206226

**Published:** 2023-07-19

**Authors:** Matthias Liebrand, Michael Rebsamen, Akari Nakamura-Utsunomiya, Luisa von den Driesch, Patrick Köck, Julien Caccia, Christoph Hamann, Roland Wiest, Michael Kaess, Sebastian Walther, Sibylle Tschumi, Takeshi Y. Hiyama, Jochen Kindler

**Affiliations:** ^1^University Hospital of Child and Adolescent Psychiatry and Psychotherapy, University of Bern, Bern, Switzerland; ^2^Support Center for Advanced Neuroimaging (SCAN), University Institute of Diagnostic and Interventional Neuroradiology, Inselspital, Bern University Hospital, University of Bern, Bern, Switzerland; ^3^Department of Medical Genetics and Pediatrics, Hiroshima University Graduate School of Biomedical and Health Sciences, Hiroshima, Japan; ^4^Department of Pediatrics, Inselspital, Bern University Hospital, University of Bern, Bern, Switzerland; ^5^Department of Child and Adolescent Psychiatry, Center for Psychosocial Medicine, University Hospital Heidelberg, Heidelberg, Germany; ^6^Translational Research Center, University Hospital of Psychiatry and Psychotherapy, University of Bern, Bern, Switzerland; ^7^Department of Integrative Physiology, Graduate School and Faculty of Medicine, Tottori University, Tottori, Japan

**Keywords:** psychosis, schizophrenia, encephalitis, autoimmune, adipsic hypernatremia, case report, catatonia

## Abstract

This is the first description of a patient in which adipsic hypernatremia, a rare autoimmune encephalitis, presented in combination with complex psychiatric symptomatology, including psychosis and catatonia. Adipsic hypernatremia is characterized by autoantibodies against the thirst center of the brain. These autoantibodies cause inflammation and apoptosis in key regions of water homeostasis, leading to lack of thirst and highly increased serum sodium. To date, the symptoms of weakness, fatigue and drowsiness have been associated with adipsic hypernatremia, but no psychiatric symptomatology. Here, we showcase the first description of an adolescent patient, in which severe and complex psychiatric symptoms presented along with adipsic hypernatremia. The patient experienced delusion, hallucinations, restlessness and pronounced depression. Further, he showed ritualized, aggressive, disinhibited and sexualized behavior, as well as self-harm and psychomotor symptoms. Due to his severe condition, he was hospitalized on the emergency unit of the child and adolescent psychiatry for 8 months. Key symptoms of the presented clinical picture are: childhood-onset complex and treatment-resistant psychosis/catatonia, pronounced behavioral problems, fatigue, absent thirst perception, hypernatremia and elevated prolactin levels. This case report renders first evidence speaking for a causal link between the autoimmune adipsic hypernatremia and the psychotic disorder. Moreover, it sheds light on a new form of autoimmune psychosis.

## Introduction

**Figure 1:**
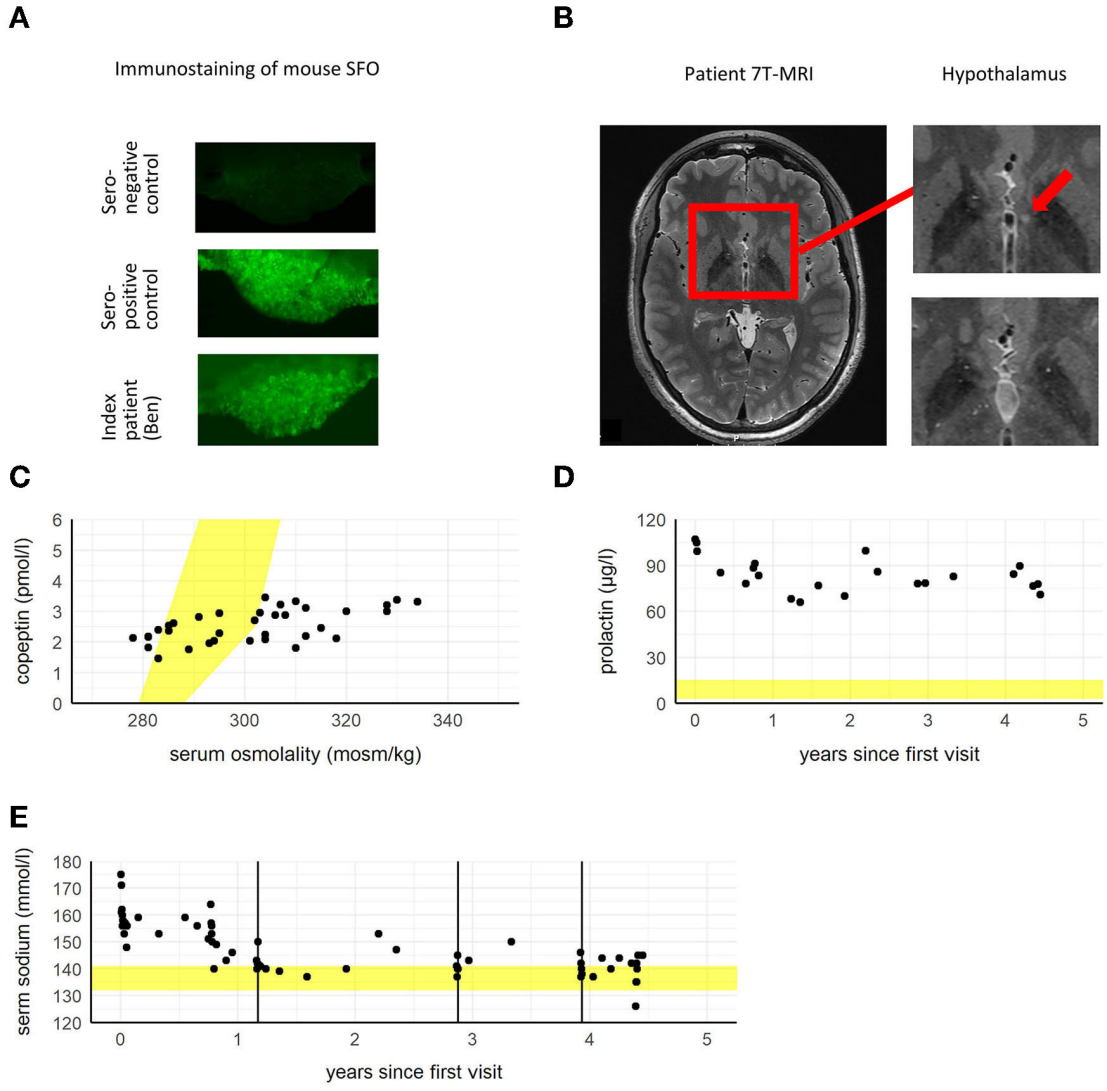
**(A)** Slice of mouse subfornical organ (SFO) tissue with control serum of a seronegative healthy control subject showing no immunological reaction in comparison to a seropositive control and to the patient's serum showing antibody binding to SFO as visible by immunostaining. **(B)** 7T-MRI showing a small T2-hyperintensity (~2 mm) in left hypothalamus close to third ventricle in the circumventricular region (left and upper right image, red arrow) 1 year after diagnosis with adipsic hypernatremia. One and a half years later the enhancement is not visible any more (lower right image). It is suspected that the hyperintensity corresponds to a lesion caused by autoantibodies, which reconstituted later on. **(C)** Serum copeptin (vasopressin marker peptide) in relation to serum osmolality. The patient's copeptin values only show minimal rise in relation to increasing serum osmolality. The expected range of copeptin values in relation to serum osmolality is marked in yellow. **(D)** Depicted is elevated serum prolactin beginning with the patient's first visit to the hospital. The physiological range of prolactin is marked in yellow. **(E)** Levels of serum sodium beginning with the patient's first visit to the hospital. The physiological range of sodium is marked in yellow. Timepoints of plasmapheresis are marked as black vertical lines.

Adipsic hypernatremia is an orphan disease, to date about 30 cases have been described worldwide (1–3). The disorder's main characteristic features are: hypernatremia without thirst sensation, dysfunctional vasopressin release, absence of structural lesions in the hypothalamic–pituitary axis otherwise explaining the clinical picture and intact urine-concentrating capacity. It is typically diagnosed in childhood, and prolactin levels are often increased. In our patient, as well as in 20 other patients diagnosed with adipsic hypernatremia, antibodies against the subfornical organ (SFO) were detected (Figure 1A) (1–3).

**Figure 2:**
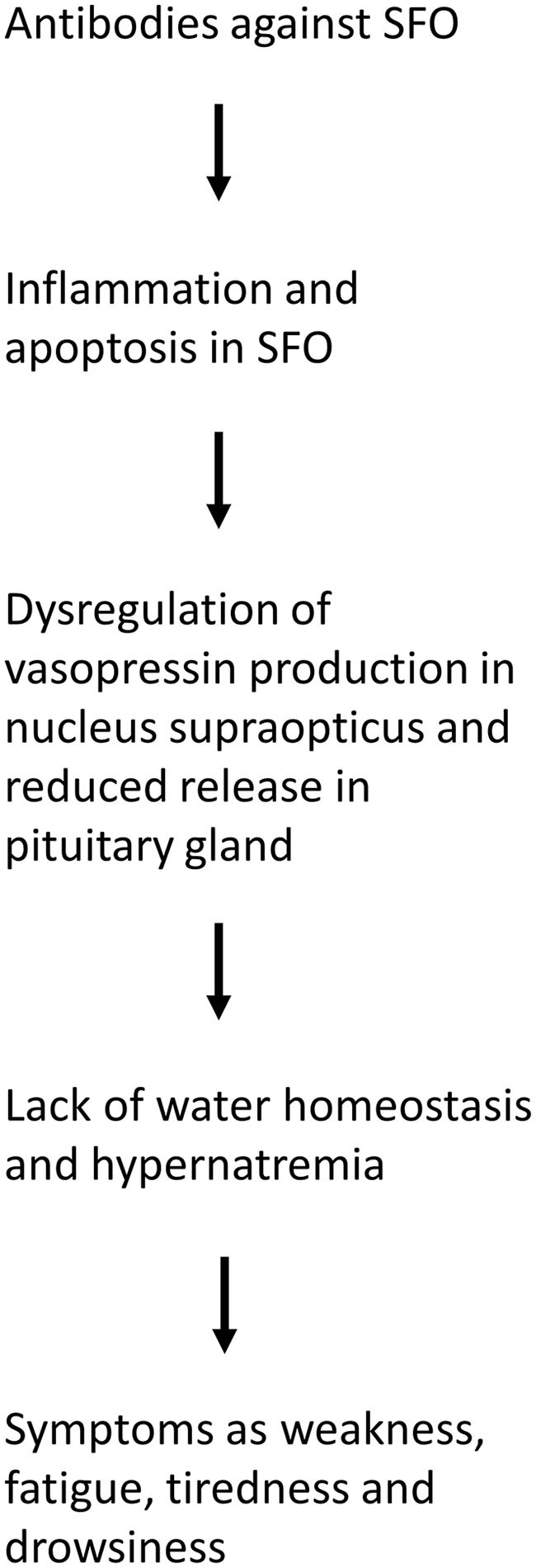
Flow diagram of the pathophysiology of adipsic hypernatremia.

The SFO, together with the organum vasculosum of the lamina terminalis (OVLT) and the median preoptic nucleus, form the key components in the regulation of thirst and fluid homeostasis (4). Importantly, the SFO hosts projections to the supraoptic nucleus, in which vasopressin is produced. Under physiological conditions, increased serum sodium is sensed in the SFO, leading to the production of vasopressin in the supraoptic nucleus. Vasopressin release in turn, results in thirst perception and increased water reabsorption in the kidneys, thereby normalizing the internal fluid status. As previously demonstrated in mouse models of adipsic hypernatremia (1), it is assumed that autoantibodies lead to inflammation and apoptosis in the SFO. As a result, sodium sensing in the SFO is impaired and levels of vasopressin remain stable irrespective of serum sodium levels. These two phenomena, autoantibodies against the SFO (Figure 1A) and dysregulation of vasopressin levels in relation to serum sodium/osmolality (Figure 1C), were observed in our patient. It can be assumed that this pathomechanism leads to dysregulation of water homeostasis, including hypernatremia and lack of thirst (Figure 2).

Adipsic hypernatremia manifests in the clinical symptoms of weakness, fatigue, tiredness and drowsiness (1) and similar symptoms have been observed in our patient. To date, no psychiatric symptomatology has been reported or associated with adipsic hypernatremia. However, our patient developed severe and complex psychiatric symptoms, including psychosis and catatonia, which will be covered in this case report.

## Case presentation

Ben (16 years, pseudonym), was admitted via the general emergency department due to logorrhea, restlessness, and severe anxiety. He had the feeling of being able to influence other people with his thoughts, heard imperative voices and was convinced he was able to restore a “magical energy” by taking a shower. Prior to admission, Ben had been experiencing these symptoms waxing and waning for several weeks. There were no major irregularities in his developmental history other than some mild features of autism in childhood (i.e., difficulty transitioning, rigidity of structure, vivid imagination). His medical history included neurodermatitis (from 4 to 18 months), conjunctivitis, asthma and attention deficit hyperactivity disorder (ADHD, treatment with 30 mg methylphenidate for several years at the time of admission). In family history no major diseases are known, other than an uncle with dysthymia/depression and asthma in Ben's father. In the pediatric university hospital, Ben was diagnosed with a polymorphic psychotic episode and treated with risperidone (3 mg/day) and lorazepam (1 mg, as needed). Within 2 weeks his condition improved and he was transferred to the emergency department of the university hospital of child and adolescent psychiatry due to behavioral problems (logorrhea, disinhibited behavior) and for optimization of pharmacological treatment. He was monitored and treated for 8 months on the emergency ward due to his broad and complex symptomatology. During this stay he was transferred to a specialized psychosis inpatient unit, but had to return to the emergency ward after about 2 weeks, because severe limitations in social functioning (disinhibited and restless behavior) prevented cohabitation with other patients.

**Figure 3:**
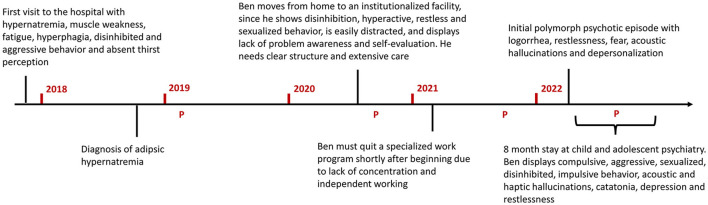
Timeline of Ben's most important medical and life events, starting with his initial visit to the pediatric university hospital. In red the years following this first visit are marked. The P's indicate timepoints of plasmapheresis. Plasmapheresis led to a replicable short-term improvement (sodium levels and water homeostasis improved directly after the first plasmapheresis; psychotic, cognitive and catatonic symptoms decreased about 3–4 months after every plasmapheresis). However, relapse/worsening of neurocognitive symptoms occurred on the long term (about 6–9 months after every plasmapheresis).

Four and a half years before this fulminant psychotic episode, Ben had been admitted to the pediatric university hospital (Figure 3) due to extreme hypernatremia (176 mmol/l, Figure 1E), a condition which, under normal circumstances is accompanied by severe symptoms, such as strong feeling of thirst, confusion, loss of appetite and cerebral bleeding.

Severe hypernatremia was discovered by his pediatrician, after Ben presented with muscle weakness and pain, in combination with general performance reduction, fatigue and hypersomnia. Ben also displayed disinhibited, persevering and aggressive behavior, motor and vocal tics and concentration difficulties. Additionally, he had gained 14 kg in 3 months due to increased appetite and showed signs of autonomic dysregulation (e.g., severe sweating, decreased cold-sensitivity). Most importantly, Ben had almost no thirst sensation. After 2 weeks, his serum sodium levels spontaneously normalized and Ben was discharged from the hospital with a prescribed drinking volume of 2 l/day. During several visits over the following year, the pediatric nephrology of the university hospital ruled out various disorders associated with hypernatremia and malfunction of water homeostasis, including salt intoxication, central and nephrogenic diabetes insipidus, limbic encephalitis, mucopolysaccharidosis, adrenoleukodystrophy and Wilson's disease. There were no findings in cerebrospinal fluid (CSF) nor in blood parameters indicating an autoimmune disorder including encephalitis [i.e., anti-N-methyl-D-aspartate (NDMA) receptor encephalitis, anti-gamma-aminobutyric acid (GABA) receptor encephalitis, systemic lupus erythematosus and others]. Also, there were no abnormalities in the electroencephalography (EEG). IQ was normal [101 as measured with Wechsler Intelligence Scale for Children (WISC-IV)], but Ben showed severe limitations in attention and was easily tired by cognitive tasks. Cognitive testing was repeated throughout the course of his treatment, revealing impairments in attention [tested with WISC-IV, Testbatterie für Aufmerksamkeitsprüfungen (TAP), Continuous Performance Test 3, (CPT 3)], learning and memory [Verbaler Lern- und Merkfähigkeitstest (VLMT), transient], social cognition [Geneva Social Cognition Scale (GeSoCS), transient], and social-emotional functioning [Intelligence and Development Scale (IDS), transient]. Parent and teacher questionnaires revealed severe abnormalities in executive functioning [Behavior Rating Inventory of Executive Function (BRIEF), Frontal Systems Behavior Scale (FrSBe)] and attention/behavior (Conners 3). Neurologic examinations did not reveal any abnormalities. In the magnetic resonance imaging (MRI) a small T2-hyperintensity of unclear etiology in the circumventricular region in hypothalamus was found, a region where part of the thirst center is located (Figure 1B). Strikingly, the release of vasopressin did not adjust to serum osmolality (Figure 1C) and prolactin levels were constantly elevated, independent of drug therapy (Figure 1D). In sum, no explanation for Ben's condition was found. Due to recurring hypernatremia (Figure 1E) and the fact that Ben was not able to maintain the prescribed daily fluid intake, a symptomatic treatment with oral desmopressin 2 x 90 μg/day was initiated. Subsequently, sodium levels stabilized in the upper normal range.

After 1 year of examination and extensive literature search, it was suspected that Ben might suffer from the very rare autoimmune adipsic hypernatremia. In an immunostaining using mouse brain tissue, it could be demonstrated that Ben's serum contains specific antibodies to the SFO (Figure 1A), confirming the diagnosis of adipsic hypernatremia.

Subsequently, he received plasmapheresis in combination with intravenous immunoglobulin therapy on a yearly basis, clearing his plasma from the autoantibodies targeting his thirst-center. Right after the first plasmapheresis cycle, it was possible to lower the dosage of desmopressin to 2 x 60 μg/day and Ben maintained normal serum sodium levels (Figure 1E). With a delay of months, the plasmapheresis had clinically significant positive effects on his psychiatric condition, as reported by him and his treatment team. This clinical improvement was objectifiable in neuropsychological testing, through significant increased performance in learning, attention and memory functions (in subtests of the WISC-IV and TAP). However, the neurocognitive improvements were short-lived and some months after each improvement Ben's symptoms worsened again. In accordance with this, the anti-SFO antibodies were no longer detectable directly after the first plasmapheresis, but reappeared later on. A treatment with steroids was discussed, but not initiated due to potential adverse psychiatric side effects (i.e., hypomania, irritability, anxiety, aggressive behavior, psychosis, cognitive deficits) and due to uncertainty about the location of the potential antigen of the SFO-antibody in the cell. In the further course Ben moved to an institutionalized group home, since his psychosocial functioning severely deteriorated and was not compatible with family life anymore. The most impairing symptoms were behavioral and emotional disinhibition, restless and sexualized behavior, impulsivity and pronounced cognitive deficits (attention deficits, lack of problem awareness, inaccurate evaluation of his behavior). Ben needed a clear daily structure and extensive care. During the following time, Ben participated in a work program specialized for disabled adolescents and adults, which was discontinued due to his attention deficits and inability to work independently. His general condition remained more or less unchanged until he presented with the polymorphic psychotic episode described above and was transferred to the child and adolescent psychiatric hospital.

**Figure 4:**
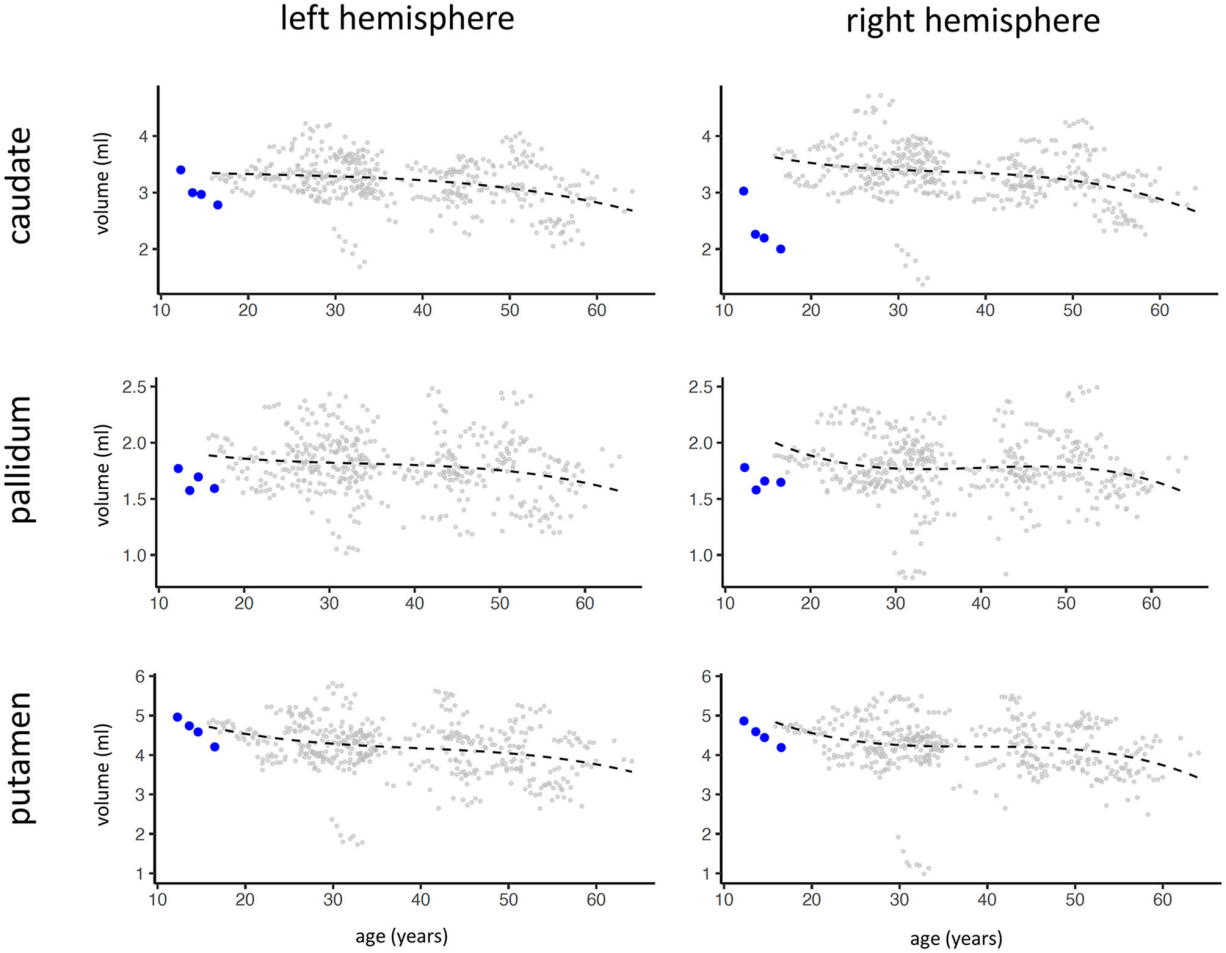
Results of volume analysis of left and right basal ganglia (caudate, pallidum, putamen) of Ben (blue dots, four contrast-enhanced MRIs have been conducted over a span of 4 years) in relation to a sample of patients with multiple sclerosis (gray dots, no healthy sample available from contrast-enhanced MRI). The black line displays the fitted mean of the multiple sclerosis patients as reference. The quantitative analysis of basal ganglia volume was performed with DL + DiReCT (7) which allows to extract brain morphometry from contrast-enhanced T1-weighted MRI (8).

During his 8-month stay on the emergency ward of the child and adolescent psychiatry, Ben displayed a broad spectrum of symptoms: motoric and behavioral compulsive rituals that severely compromised cohabitation with others (e.g., urinating in drinking bottles, collecting and hoarding trash); sudden and mostly unexplained aggressive outbursts (e.g., attacking other patients); sexualized and disinhibited behavior (reduced social inhibition, sexualized talk and actions); and impulsive self-harming behavior (e.g., swallowing a pen, backflip with clavicula fracture). Ben displayed a nearly constant restlessness, often pacing from one side of the ward to the other. Further, he experienced auditory hallucinations (male voices), severely depressed mood and desperation including suicidal thoughts. Additionally, he showed psychomotor symptoms including reduced mimics, motoric slowing, reduced speech modulation, decrease of verbal output, response latency, thought blocking, rigor and ambitendency. These signs qualify for the syndrome of catatonia (5, 6), as do compulsive rituals, restlessness, pacing and impulsive attacks of others. For the treatment of catatonia, benzodiazepines (lorazepam up to 5 mg/day) were prescribed for a period of almost 2 weeks. However, this medication was discontinued due to limited effect on catatonic symptoms and because of subjective negative side effects on cognition. Multiple antipsychotics were prescribed, including risperidone, levomepromazine, haloperidol and zuclopenthixol, none of which led to a fundamental change in Ben's condition. However, clozapine proved to be the most effective (current dose is 100 mg/day, plasma levels were in therapeutic range at discharge). Additionally, fluvoxamine (200 mg/day) was prescribed, to treat his depressive and ritualized symptomatology. In the university hospital, MRIs (two 7T-, four 3T- and two 1,5T-MRIs) were regularly performed as longitudinal follow-up. Structural analysis of contrast-enhanced MRIs showed a substantial longitudinal decrease of gray matter volume in bilateral basal ganglia (Figure 4).

Over the course of his eight-month treatment period, Ben's condition gradually and slightly improved. Plasmapheresis led to short-term improvements, however without sustainable long-term effects. Under treatment with clozapine (last blood level 133 μmol/l), Ben's psychotic symptoms improved and in particular hallucinations and delusions disappeared. Ritualized behavior improved in response to medication with fluvoxamine and aggressive outbursts reduced significantly, however, not reaching full remission. After 8 months, Ben's condition allowed him to be discharged from the psychiatric emergency ward. An experimental therapy with rituximab (anti-CD20 antibody) is about to be started. Currently, he lives in a specialized social education institution and will likely need extensive care and psychiatric treatment in the future.

## Discussion

To date, Ben is the first patient in whom polymorphic psychosis and catatonia in combination with adipsic hypernatremia have been described. In the following, we will first argue why psychotic symptoms in the presented case are not related to (a) another autoimmune encephalitis, to (b) schizophrenia without organic reason or to (c) another psychiatric differential diagnosis associated with psychosis. Then, we will turn to arguments for a causal role of adipsic hypernatremia regarding the psychotic and catatonic symptoms of the patient. Finally, we will discuss possible pathophysiological mechanisms.

Another encephalitis seems very unlikely as there were no findings in CSF, EEG, nor in blood tests, indicating brain inflammation (see above). The broad symptom spectrum including symptoms mimicking frontal lobe dysfunction (i.e., disinhibition, sexualized behavior, cognitive deficits) in combination with pharmacological treatment resistance and young age speak against schizophrenia without organic reason. Finally, we believe that the main other psychiatric differential diagnoses in this case are highly unlikely or can be ruled out: bipolar disorder (no episodic changes of the mood, no phases of elevated mood/mania), schizotypal personality disorder (no social anxiety and rather disinhibited behavior, no unconventional beliefs, no paranoia), depression (depression was not the key symptom and only one of many, depression occurred after the first psychotic episode), PTSD (no trauma, no flashbacks), borderline personality disorder (no instable relationships/self-image, no identity problems, no affective instability) and dissociative disorder (no dissociations).

Based on the presented single case, we are not able to prove a causal connection between underlying adipsic hypernatremia and following psychosis/catatonia. However, several arguments speak for a causal relation of adipsic hypernatremia and his psychiatric condition. First, adipsic hypernatremia evolved before the first psychotic episode, allowing a potential causal role in manifestation of later psychosis. Second, Ben displayed a symptomatology typical for immune-mediated schizophreniform psychosis (9): acute onset of polymorphic psychotic symptoms, catatonia, speech problems (absent modulation, response latency), autonomic dysregulation (e.g., severe sweating, decreased cold-sensitivity) and autoimmune predisposition (atopic dermatitis in early childhood, conjunctivitis, asthma). The most common type of autoimmune-mediated psychosis is anti-NMDA receptor encephalitis, with which Ben shows a striking overlap in symptomatology (10): predominance in child- and young adulthood, hypersomnia, hyperphagia, excitement, disinhibition, agitation, aggression, rapid onset of psychotic symptoms, catatonia, both negative and positive symptoms at first presentation and decreased verbal output. Third, his response to antipsychotic treatment remained very limited, a common observation in autoimmune-related psychosis (11). Fourth, several psychiatric disorders, including autism spectrum disorder, depression and schizophrenia have been found to share the same neuroinflammatory pathways (12), which is closely in line with Ben's symptom range. Fifth and most importantly, Ben's water homeostasis and serum sodium levels, simultaneously with his psychiatric/psychotic symptomatology, improved after plasmapheresis. Plasmapheresis was performed on a yearly basis and after each treatment, Ben's cognitive and psychiatric symptoms improved and sodium levels normalized.

Currently, this is the first and only case of psychosis/schizophrenia combined with adipsic hypernatremia. However, adipsic hypernatremia is a poorly understood disorder, in which research is still in a very early phase, with only a few published case reports. Moreover, most reported patients were children, and schizophrenia rarely emerges during childhood (13, 14).

This case raises the question of the link between adipsic hypernatremia and psychotic symptomatology. One possible mechanism could be mediated via vasopressin, which is fundamentally dysregulated in adipsic hypernatremia. Vasopressin-deficient rats have been shown to be a useful model for schizophrenia (15). Also, studies reported decreased blood levels (16, 17) and intracranial gene expression (18) of vasopressin in schizophrenia patients. Moreover, intranasal vasopressin seems to have a beneficial effect on symptomatology of schizophrenia (19). Another link might be dopamine, possibly the key neurotransmitter in neuropathology of schizophrenia. Although we have no knowledge of Ben's dopamine levels, a constant elevation of prolactin was observed. Prolactin is closely interlinked with dopamine, given dopamine suppresses the release of prolactin. Also, in another patient with adipsic hypernatremia, a prolactin stimulation test using metroclopramide did not result in excess of prolactin, suggesting disturbed dopamine baseline levels [patient covered in (3)]. Notably, basal ganglia atrophy was detected in Ben's MRI (Figure 4), where dopamine is a key neurotransmitter. Importantly, basal ganglia (beside hypothalamus and pituitary gland) host the specific antigen to which autoantibodies in adipsic hypernatremia bind (manuscript under preparation). This suggests, that in Ben's case, antibodies and related encephalitis lead to apoptosis and atrophy in crucial brain regions including basal ganglia, thereby dysregulating key neurotransmitters as vasopressin and dopamine. We assume that this pathophysiology fundamentally disrupts processes in the basal ganglia and in the motor system and thereby leads to psychotic symptomatology and catatonia.

In conclusion, we present a novel case of autoimmune related psychosis with autoantibodies against the SFO. The main characteristic features of this condition which might guide the clinician are: childhood-onset complex and treatment-resistant psychosis and catatonia in combination with severe behavioral problems, fatigue, absent thirst, hypernatremia and elevated prolactin. Many unknowns concerning the links between adipsic hypernatremia and psychosis remain and causal connections have yet to be established. Nevertheless, we present strong indications for a causal link. Autoimmune psychosis has gained increasing interest in recent years (20), and the present case opens a new perspective on antibody related autoimmune psychosis.

## Data availability statement

The original contributions presented in the study are included in the article, further inquiries can be directed to the corresponding author.

## Ethics statement

Ethical review and approval was not required for the study on human participants in accordance with the local legislation and institutional requirements. Written informed consent to be described in this case report was provided by the participants' legal guardian/next of kin. Written informed consent was obtained from the minor(s)' legal guardian/next of kin for the publication of any potentially identifiable images or data included in this article. Written informed consent was obtained from the patient and his parents for the publication of this case report.

## Author contributions

ML: investigation, validation, data curation, visualization writing—original draft, and writing—review and editing. MR: methodology, software, formal analysis, and writing—review and editing. AN-U, LD, and PK: writing—review and editing. JC: investigation. CH: conceptualization and writing—review and editing. RW: resources. MK: resources and writing—review and editing. SW: investigation and writing—review and editing. ST: investigation, resources, and writing—review and editing. TH: resources, methodology, and writing—review and editing. JK: supervision, project administration, resources, and writing—review and editing. All authors contributed to the article and approved the submitted version.
